# Staged Carotid Endarterectomy Following Percutaneous Transluminal Angioplasty for Hemodynamically Compromised Internal Carotid Artery Stenosis at High Risk of Hyperperfusion: A Case Report

**DOI:** 10.7759/cureus.109130

**Published:** 2026-05-18

**Authors:** Yutaka Fuchinoue, Yuki Sakaeyama, Ryo Matsuzaki, Masaaki Nemoto, Nobuo Sugo

**Affiliations:** 1 Department of Neurosurgery, Toho University, Chiba, JPN; 2 Department of Neurosurgery, Toho University, Tokyo, JPN

**Keywords:** carotid artery stenosis, carotid endarterectomy (cea), cerebral hyperperfusion syndrome, cerebral perfusion imaging, magnetic resonance perfusion imaging, ocular ischemic syndrome, staged angioplasty

## Abstract

Cerebral hyperperfusion syndrome (CHS) is a rare but potentially devastating complication after carotid revascularization in patients with severe carotid stenosis and impaired cerebral hemodynamics. We report a case of staged carotid endarterectomy (CEA) performed to reduce the risk of postoperative CHS.

A 78-year-old woman underwent ophthalmologic evaluation for decreased vision in the left eye and was referred to our department because ocular ischemia related to ipsilateral carotid artery disease was suspected. Carotid ultrasonography demonstrated severe stenosis of the left internal carotid artery (ICA), with a peak systolic velocity of 353 cm/second and 87% stenosis by the area method, along with marked calcification and plaque ulceration. Digital subtraction angiography revealed near-occlusive left ICA stenosis with delayed antegrade flow, 99% stenosis by the North American Symptomatic Carotid Endarterectomy Trial (NASCET) method, occlusion of the left external carotid artery, and poor collateral circulation. Magnetic resonance angiography (MRA), arterial spin labeling (ASL), and N-isopropyl-p-[123I]-iodoamphetamine single-photon emission computed tomography (IMP-SPECT) demonstrated left hemispheric hemodynamic compromise.

Because the patient was considered to be at increased risk for postoperative CHS and the lesion was heavily calcified, staged CEA consisting of percutaneous transluminal angioplasty (PTA) followed by delayed CEA was selected. After PTA, residual stenosis improved to 85% by the NASCET method and 83% by the area method, without new ischemic lesions. MRA, ASL, and IMP-SPECT showed improved left hemispheric perfusion. Seven days later, CEA was performed without CHS or major perioperative complications. The patient was discharged home 10 days after CEA with a modified Rankin Scale score of 0, and no restenosis or neurological complications had occurred at three years of follow-up.

Staged CEA after PTA may be an individualized treatment option for selected patients with severe carotid stenosis, marked hemodynamic compromise, and lesion morphology that favors plaque removal over primary stenting.

## Introduction

Cerebral hyperperfusion syndrome (CHS) is an uncommon but potentially devastating complication after carotid revascularization because it can lead to intracranial hemorrhage and severe neurological deterioration [1]. Impaired cerebrovascular reserve and abrupt restoration of flow after revascularization are considered central mechanisms in the development of CHS [2]. For carotid artery stenting (CAS), staged angioplasty, consisting of limited balloon angioplasty followed by delayed definitive revascularization, has been investigated as a strategy to reduce this risk by allowing gradual reperfusion [3-5]. In the randomized STEP trial, the incidence of the hyperperfusion phenomenon was significantly lower in the staged angioplasty group [6]. However, when plaque morphology makes definitive plaque removal preferable to primary stenting, the optimal strategy is less well established [7,8]. Only a limited number of reports have described a stepwise approach in which initial percutaneous transluminal angioplasty (PTA) is used to achieve gradual reperfusion before delayed carotid endarterectomy (CEA) [7,8].

We report a patient with severe internal carotid artery stenosis, marked ipsilateral hemodynamic compromise, and imaging features suggestive of a hard calcified plaque who was successfully treated with staged CEA consisting of initial PTA followed by delayed CEA.

## Case presentation

A 78-year-old woman underwent ophthalmologic evaluation for decreased vision in the left eye two months before PTA. Decimal visual acuity was 1.0p in the right eye and 0.8 without correction in the left eye. Intraocular pressure measured by noncontact tonometry was 18 mmHg in the right eye and 15 mmHg in the left eye. Optical coherence tomography and optical coherence tomography angiography showed no major abnormalities. Fluorescein angiography showed findings suggestive of left retinal periphlebitis. Laser speckle flowgraphy demonstrated reduced retinal and choroidal circulation in the left eye compared with the right eye. Based on these ophthalmologic findings, ocular ischemia related to ipsilateral carotid artery disease was diagnosed, and she was referred to our department for further evaluation.

Her pretreatment modified Rankin Scale score was 0 [9]. She was never a smoker. Her medical history included a prior myocardial infarction treated with percutaneous coronary intervention. She had been taking aspirin 100 mg once daily, which was continued throughout the perioperative period. She had no history of hypertension, diabetes mellitus, dyslipidemia, atrial fibrillation, or chronic kidney disease. Neurological examination before PTA showed no focal neurological deficits, including motor weakness, sensory disturbance, aphasia, or neglect, and the National Institutes of Health Stroke Scale score was 0 [10].

**Figure 1:**
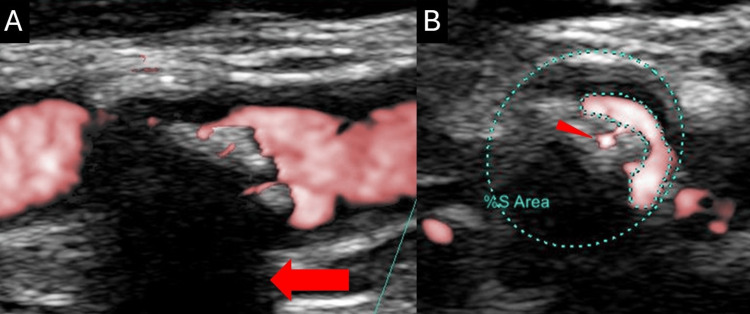
Preoperative carotid ultrasonography findings (A) Color Doppler carotid ultrasonography showing marked calcification with acoustic shadowing at the stenotic lesion (arrow). (B) Color Doppler carotid ultrasonography demonstrating plaque ulceration (arrowhead)

Carotid ultrasonography demonstrated severe stenosis at the origin of the left ICA, with a peak systolic velocity of 353 cm/second and 87% stenosis by the area method. The lesion showed marked calcification with acoustic shadowing and plaque ulceration on color Doppler imaging (Figures 1A, 1B).

**Figure 2:**
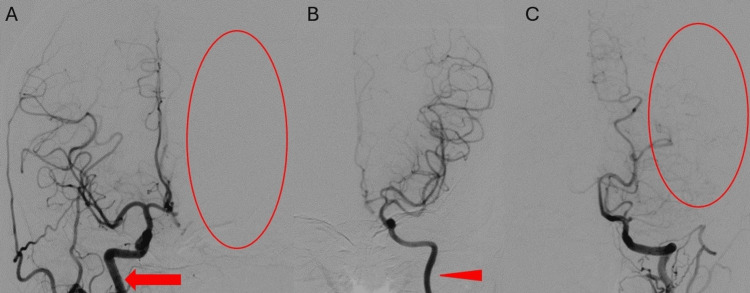
Preoperative cerebral angiographic findings (A) Frontal view of right common carotid angiography showing prominent visualization of the internal carotid artery (arrow). No collateral perfusion to the left hemisphere through the anterior communicating artery is identified (oval). (B) Frontal view of left common carotid angiography showing that the left internal carotid artery is visualized as thinner than the right internal carotid artery (arrowhead). (C) Frontal view of left vertebral angiography showing minimal collateral perfusion to the left middle cerebral artery territory through leptomeningeal anastomosis from the posterior cerebral artery (oval)

**Figure 3:**
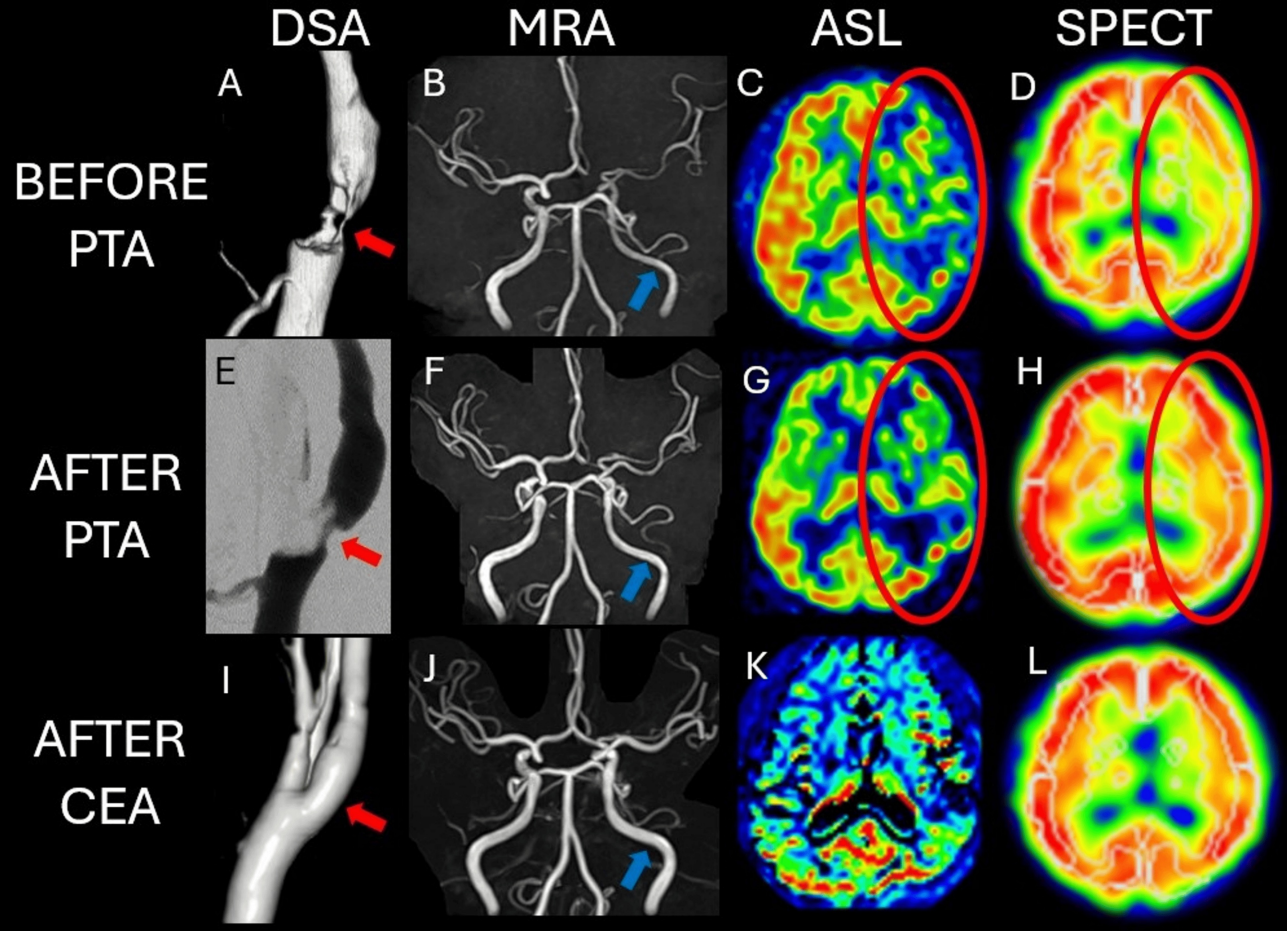
Serial multimodal imaging before and after staged treatment (A) Preoperative three-dimensional rotational angiography showing pseudo-occlusion of the left internal carotid artery (red arrow). (B) Preoperative MRA showing decreased signal intensity in the left internal carotid artery system (blue arrow). (C) Preoperative ASL showing extensive hypoperfusion in the left cerebral hemisphere (oval). (D) Preoperative IMP-SPECT showing reduced perfusion in the left cerebral hemisphere corresponding to the ASL findings (oval). (E) Lateral view of common carotid angiography after PTA showing sufficient luminal expansion compared with the pretreatment state (red arrow). (F) MRA after PTA showing improved signal intensity in the left internal carotid artery system (blue arrow). (G) ASL after PTA showing improvement of perfusion in the left cerebral hemisphere compared with the pretreatment study (oval). (H) IMP-SPECT after PTA showing improvement of perfusion in the left cerebral hemisphere compared with the pretreatment study (oval). (I) Postoperative three-dimensional computed tomography angiography after CEA showing resolution of the stenotic lesion (red arrow) and recanalization of the external carotid artery system. (J) Postoperative MRA showing that the left internal carotid artery system (blue arrow) has a caliber and signal intensity comparable to those of the right side. (K) Postoperative ASL showing no laterality of cerebral blood flow. (L) Postoperative IMP-SPECT showing no laterality of cerebral blood flow IMP-SPECT: N-isopropyl-p-[123I]-iodoamphetamine single-photon emission computed tomography; ASL: arterial spin labeling; MRA: magnetic resonance angiography; CEA: carotid endarterectomy; PTA: percutaneous transluminal angioplasty

Digital subtraction angiography revealed near-occlusive stenosis of the left ICA with markedly delayed antegrade flow, and the pre-PTA stenosis rate was 99% by the North American Symptomatic Carotid Endarterectomy Trial (NASCET) method. The left external carotid artery was occluded. On frontal right common carotid angiography, the right ICA was prominently visualized, and no collateral perfusion to the left hemisphere through the anterior communicating artery was identified (Figure 2A). On frontal left common carotid angiography, the left ICA was visualized as thinner than the right ICA (Figure 2B). Left vertebral angiography showed only minimal collateral perfusion to the left middle cerebral artery territory through leptomeningeal anastomosis from the posterior cerebral artery (Figure 2C). Three-dimensional rotational angiography before treatment showed pseudo-occlusion of the left ICA (Figure 3A).

At the time of neurosurgical evaluation, the patient had already been diagnosed with ocular ischemia based on ophthalmologic findings. Therefore, the neurosurgical diagnostic evaluation focused on the underlying carotid artery lesion and cerebral hemodynamic status. Regarding the carotid lesion, atherosclerotic stenosis was considered the most likely diagnosis because of the typical plaque morphology, marked calcification, and absence of a history of cervical irradiation. There were no findings suggestive of carotid dissection or vasculitis.

Magnetic resonance angiography (MRA) demonstrated reduced signal intensity in the left ICA system, and ASL, as well as N-isopropyl-p-[123I]-iodoamphetamine single-photon emission computed tomography (IMP-SPECT), showed extensive hypoperfusion in the left cerebral hemisphere (Figures 3B-3D). Quantitative SPECT showed cerebral blood flow values of 25.42 on the right and 22.73 on the left, indicating that cerebral blood flow on the left side was approximately 10.6% lower than that on the right. Cerebrovascular reserve testing with acetazolamide challenge was not performed because of concern that it could provoke cerebral ischemia in the setting of severe hemodynamic compromise; therefore, quantitative cerebrovascular reserve values were not obtained.

Given the chronic near-occlusive lesion, poor collateral circulation, and reduced ipsilateral cerebral perfusion, the risk of CHS after abrupt single-stage revascularization was considered high. Because the plaque was also heavily calcified and ulcerated, staged CEA consisting of initial PTA followed by delayed CEA was selected.

PTA was performed as the first-stage procedure while aspirin monotherapy was continued. The aim of this first-stage procedure was not to achieve complete lesion expansion but to gradually restore antegrade flow. After sheath insertion, 5,000 units of intravenous heparin was administered with a target activated clotting time of >250 seconds, although activated clotting time was not measured during the procedure.

Because the lesion was extremely narrow, it was first crossed using a Synchro STANDARD 0.014-inch, 215-cm guidewire (Stryker Neurovascular, Fremont, CA) with an Excelsior SL-10 microcatheter (Stryker Neurovascular). The guidewire was then exchanged for a Synchro SUPPORT 0.014-inch, 300-cm guidewire (Stryker Neurovascular), and the Excelsior SL-10 microcatheter was removed. Predilation was performed using a Gateway balloon measuring 2.0 × 20 mm (Stryker Neurovascular), inflated at the nominal pressure for 30 seconds. A SpiderFX distal filter protection device (Medtronic, Minneapolis, MN) was then deployed distal to the lesion. Subsequent balloon angioplasty was performed using a 3.0 × 20 mm Sterling balloon (Boston Scientific, Marlborough, MA), inflated at the nominal pressure for 30 seconds. The purpose of PTA was limited luminal expansion to improve antegrade flow rather than complete revascularization.

After PTA, residual stenosis improved to 85% by the NASCET method and 83% by the area method. Lateral common carotid angiography demonstrated sufficient luminal expansion compared with the pretreatment state (Figure 3E). Doppler ultrasonography after PTA showed an increase in the peak systolic velocity at the stenotic segment to 3.8 m/s (380 cm/sec). Although PTA usually reduces flow velocity across a stenotic lesion, the increase in velocity in this patient was interpreted as reflecting improved antegrade flow because the pretreatment lesion had near-occlusive morphology with markedly reduced flow. No new ischemic lesions were detected after PTA.

After PTA, the patient was managed in the intensive care unit for neurological observation and blood pressure control, with a target systolic blood pressure of <120 mmHg. Surveillance for CHS was performed using serial neurological assessment, MRI ASL, and IMP-SPECT. Diffusion-weighted imaging (DWI), MRA, ASL, and IMP-SPECT were obtained several days after PTA. MRA after PTA showed improvement of signal intensity in the left ICA system (Figure 3F), and ASL and IMP-SPECT showed no apparent laterality of cerebral blood flow after PTA (Figures 3G, 3H).

**Figure 4:**
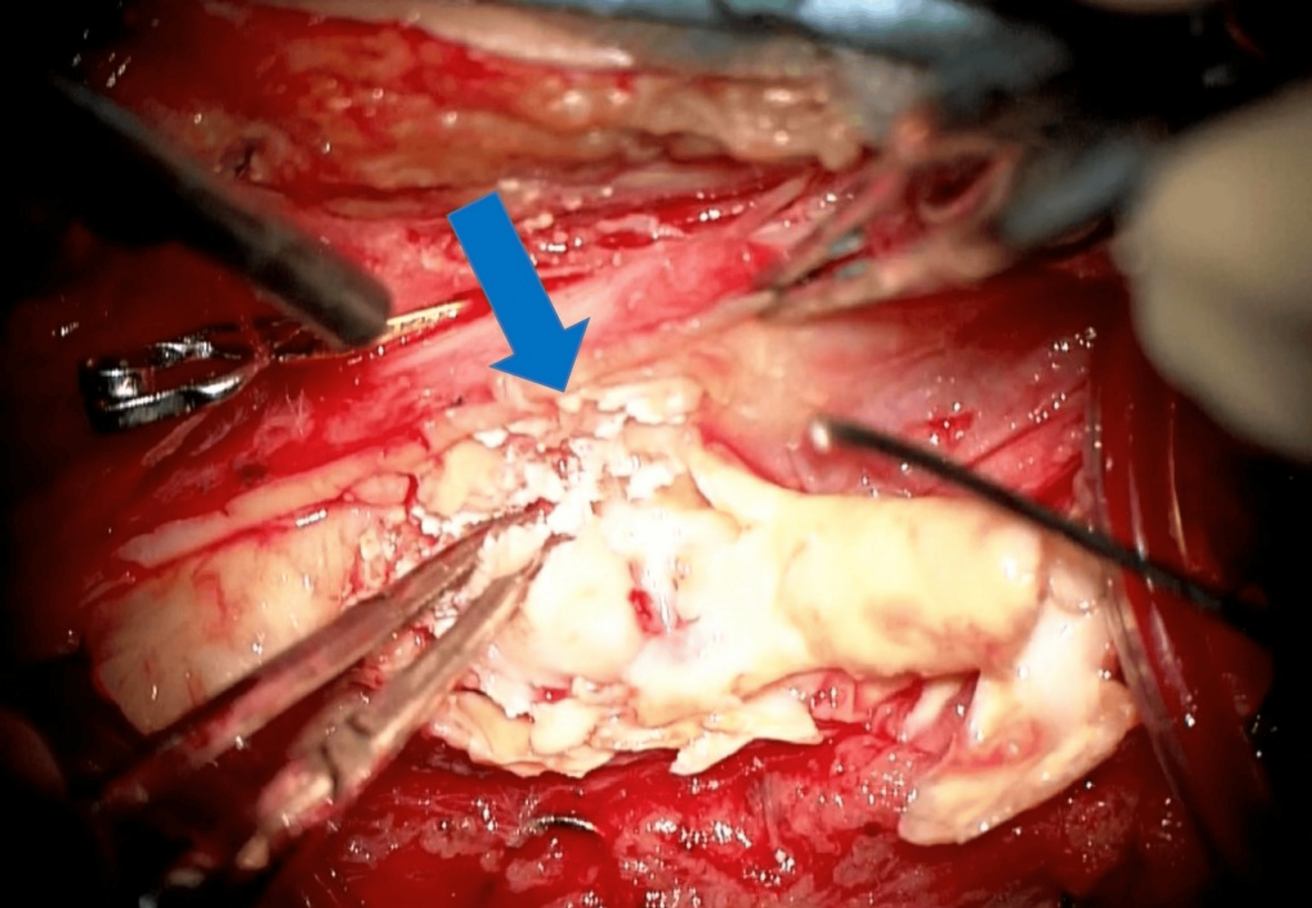
Intraoperative photograph obtained during carotid endarterectomy The plaque shows severe calcification (arrow)

Because the postoperative course after PTA was stable, CEA was performed seven days later under general anesthesia. During CEA, an intraluminal shunt was used. Patch angioplasty was not performed, and the arteriotomy was closed primarily. Intraoperative neurophysiological monitoring was performed using somatosensory evoked potentials and motor evoked potentials. The target systolic blood pressure during and after CEA was <120 mmHg. Intraoperatively, the excised plaque showed marked calcification, consistent with the preoperative imaging findings (Figure 4). The patient showed no new neurological deficits after CEA.

After CEA, the patient was managed in the intensive care unit for neurological observation and blood pressure control. Surveillance for CHS was performed using serial neurological assessment, MRI ASL, and IMP-SPECT. Wound observation and neurological examination were performed to detect perioperative complications. No new neurological deficits, wound complications, or findings suggestive of CHS were observed.

Postoperative three-dimensional computed tomography angiography demonstrated resolution of the stenotic lesion and recanalization of the external carotid artery system (Figure 3I). Follow-up MRA after CEA demonstrated that the left ICA system had a caliber and signal intensity comparable to those of the right side (Figure 3J). Postoperative ASL and IMP-SPECT showed no laterality of cerebral blood flow after CEA (Figures 3K, 3L).

Cognitive screening also improved during the staged treatment course. The Mini-Mental State Examination score improved from 28 before PTA to 30 after CEA [11], and the Frontal Assessment Battery score improved from 14 before PTA to 17 after CEA [12]. The patient was discharged home 10 days after CEA with a modified Rankin Scale score of 0. Aspirin monotherapy was continued after discharge. At three years of follow-up, no restenosis or neurological complications had occurred.

## Discussion

CHS is an uncommon but potentially devastating complication after carotid revascularization, particularly when intracranial hemorrhage occurs, and this makes preventive strategies clinically important [1]. In the present case, chronic near-occlusive carotid stenosis, poor collateral circulation, reduced baseline perfusion, and the expected abrupt increase in flow after definitive revascularization led us to consider the patient to be at increased risk for postoperative hyperperfusion. Cerebrovascular reserve testing with acetazolamide challenge was not performed because of concern that it could provoke cerebral ischemia in the setting of severe hemodynamic compromise. Therefore, the hemodynamic risk assessment was based on the combination of angiographic collateral findings and perfusion imaging findings, including ASL and IMP-SPECT.

The quantitative stenosis and flow-velocity findings in this case should be interpreted in the context of near-occlusive or pseudo-occlusive morphology. Before PTA, carotid ultrasonography showed 87% stenosis by the area method and a peak systolic velocity of 353 cm/second, whereas angiographic assessment showed 99% stenosis by the NASCET method. After PTA, residual stenosis was 83% by the area method and 85% by the NASCET method, and Doppler ultrasonography showed an increase in peak systolic velocity to 3.8 m/s. In ordinary stenotic lesions, PTA generally reduces flow velocity across the stenosis; however, in this near-occlusive lesion, the post-PTA increase in velocity was interpreted as reflecting improved antegrade flow through a previously critically narrowed segment rather than worsening stenosis. Therefore, the purpose of PTA was limited luminal expansion and gradual reperfusion, not complete revascularization.

For CAS, staged angioplasty was introduced to allow gradual reperfusion, and subsequent studies have reported acceptable safety and a lower frequency of hyperperfusion-related events or surrogate hyperperfusion findings in selected high-risk patients [3-5]. In the randomized STEP trial, the incidence of the hyperperfusion phenomenon was significantly lower in the staged angioplasty group [6]. Accordingly, staged angioplasty should be interpreted as a strategy to attenuate hyperperfusion-related hemodynamic stress rather than as a definitively established standard for all high-risk lesions [5,6].

Our case differed from conventional staged CAS because stenting was considered suboptimal in the setting of a hard, calcified plaque with acoustic shadowing and ulceration, where incomplete expansion and plaque-related embolic concerns were relevant. Stepwise revascularization by CEA after PTA has been reported only rarely [7,8]. Egashira et al. described stepwise revascularization by CEA after balloon angioplasty for symptomatic severe carotid artery stenosis [7]. Marutani et al. later reported CEA following PTA for a severely calcified carotid plaque with hemodynamic impairment to prevent hyperperfusion [8].

Similarly, Yoshimoto et al. described a stepwise strategy in which superficial temporal artery-middle cerebral artery bypass was performed first, and CEA was added weeks later to achieve gradual restoration of cerebral blood flow and prevent postoperative hyperperfusion [13]. However, a bypass-first strategy is more invasive than endovascular predilation, and in the present case, its applicability was limited because the ipsilateral external carotid artery was occluded. Taken together, these reports support the broader principle that a gradual increase in cerebral perfusion before definitive CEA may be useful in selected patients at high risk for CHS [7,8,13]. A dedicated literature review table summarizing prior similar reports and the present case is provided in Table 1.

**Table 1:** Prior reports of staged or stepwise revascularization before definitive CEA CAS: carotid artery stenting; CEA: carotid endarterectomy; CHS: cerebral hyperperfusion syndrome; ECA: external carotid artery; ICA: internal carotid artery; IMP SPECT: N-isopropyl-p-[123I]-iodoamphetamine single-photon emission computed tomography; mRS: modified Rankin Scale; NASCET: North American Symptomatic Carotid Endarterectomy Trial; PTA: percutaneous transluminal angioplasty; STA-MCA: superficial temporal artery to middle cerebral artery

Study	Design/cases	Patient(s)	Presentation	Lesion/key risk features	Hemodynamic assessment	First-stage procedure	Interval to CEA	Definitive treatment	Outcome/CHS
Yoshimoto et al. [13]	Case series; 4 patients	Individual demographics not specified in accessible abstract	Severe cerebral hypoperfusion in patients for whom CEA was indicated	Extremely severe ipsilateral or bilateral carotid stenosis; severe cerebral hypoperfusion; poor cerebrovascular reserve	Perfusion studies were used to evaluate cerebral blood flow and reserve	STA-MCA bypass	A few weeks	CEA	Good results without postoperative problems reported; no postoperative hyperperfusion syndrome described in the accessible abstract
Egashira et al. [7]	Case report; 1 patient	68-year-old man	Left hemiparesis and dysarthria	Severe right cervical ICA stenosis; vulnerable plaque components; high risk for postoperative hyperperfusion and ischemic complications after CAS	Preoperative cerebral blood flow evaluation and plaque characterization	PTA	15 days	CEA	Postoperative SPECT showed no hyperperfusion; no neurological symptoms after either procedure
Marutani et al. [8]	Case report; 1 patient	77-year-old woman	Severe proximal left ICA stenosis	Severe circular calcification; hemodynamic impairment	123I-IMP SPECT showed reduced cerebral blood flow and reduced cerebrovascular reserve in the left hemisphere; SPECT 2 days after PTA showed improved cerebral blood flow	PTA	7 days	CEA	Postoperative IMP-SPECT showed improved cerebral blood flow without hyperperfusion; discharged without neurological deficits
Present case	Case report; 1 patient	78-year-old woman	Decreased left vision/ocular ischemia	Near-occlusive left ICA stenosis; left ECA occlusion; poor collateral circulation; marked calcification and plaque ulceration; pre-PTA stenosis 99% by NASCET and 87% by area method	ASL and IMP-SPECT showed left hemispheric hypoperfusion; quantitative SPECT CBF was 25.42 on the right and 22.73 on the left; acetazolamide challenge was not performed and quantitative CVR values were not obtained	PTA	7 days	CEA	No CHS or new neurological deficit; discharged home 10 days after CEA with mRS 0; no restenosis or neurological complications at 3 years

In the present patient, PTA was completed without new ischemic lesions, and delayed CEA was completed without CHS while perfusion imaging and bedside cognitive screening improved. This favorable course suggests that initial limited PTA may have allowed partial hemodynamic adaptation before definitive plaque removal by CEA. However, the improvement in Mini-Mental State Examination and Frontal Assessment Battery scores should be interpreted cautiously because practice effects or spontaneous variability cannot be excluded. Although the optimal indications, interval between stages, and hemodynamic thresholds for staged CEA remain to be clarified, this strategy may represent a promising treatment option for selected patients with severe carotid stenosis and a high risk of CHS [7,8,13]. Additional reports have emphasized that reduced cerebrovascular reserve, marked postprocedural flow increases, and postoperative hypertension remain central considerations in the prediction and prevention of CHS [14-17].

This case has several limitations. First, cerebrovascular reserve testing with acetazolamide challenge was not performed because of concern that it could provoke cerebral ischemia in the setting of severe hemodynamic compromise; therefore, quantitative cerebrovascular reactivity (CVR) values were not obtained. Accordingly, the assessment of hyperperfusion risk was based on chronic near-occlusive stenosis, poor collateral circulation, reduced ipsilateral cerebral perfusion on ASL and IMP-SPECT, and the expected abrupt increase in flow after revascularization, rather than on quantitative CVR testing. Second, although both ultrasound-based area stenosis and angiographic NASCET measurements were provided before and after PTA, these values should be interpreted in the context of near-occlusive or pseudo-occlusive morphology. Third, this is a single case, and the optimal degree of initial balloon dilatation, interval before CEA, and objective hemodynamic criteria for selecting staged CEA remain uncertain. Further accumulation of similar cases is needed to clarify the role of this strategy.

## Conclusions

Staged CEA, consisting of initial PTA followed by delayed CEA, may be an individualized treatment option for selected patients with severe carotid stenosis who have marked hemodynamic compromise and lesion morphology that favors plaque removal over primary stenting.

In the present patient, initial limited PTA appeared to allow gradual reperfusion before definitive plaque removal by CEA, and CHS did not occur. Because acetazolamide challenge testing was not performed due to concern for provoking cerebral ischemia and quantitative CVR values were not obtained, the indication for staged treatment in this case should be interpreted as individualized and hypothesis-generating rather than definitive. Further accumulation of similar cases is needed to clarify the optimal indications, degree of initial balloon dilatation, and timing of delayed CEA.
